# Preparing for tomorrow: Iranian medical students’ attitudes toward climate change and its integration into the medical curriculum

**DOI:** 10.1186/s12909-026-09093-y

**Published:** 2026-03-31

**Authors:** Noushin Kohan, Zahra Karimian, Soleiman Ahmady, Shima Aliebrahimi, Hoda Ahmari Tehran, Manijeh Hooshmandja

**Affiliations:** 1 Department of Medical Education and eLearning in Medical Education, Smart University of Medical Sciences, Tehran, Iran; 2 Department of e-Learning in Medical Sciences, Virtual School and Center of Excellence in e-Learning, Shiraz University of Medical Sciences, Shiraz, Iran; 3 School of Medical Education and Learning Technology, Shahid Beheshti University of Medical Sciences, Tehran, Iran; 4 Department of LIME, Research Affiliated Faculty, Karolinska Institute, Solna, Sweden; 5 Department of Artificial Intelligence, Smart University of Medical Sciences, Tehran, Iran; 6 Spiritual Health Research Center, Qom University of Medical Sciences, Qom, Iran

**Keywords:** Climate Change, Attitude, Curriculum, Medical Students

## Abstract

**Background:**

Climate change is widely recognized as one of the most significant threats to global health today. Medical doctors play a crucial role in climate change mitigation and adaptation by educating patients, advocating for sustainable healthcare practices, and incorporating climate-related health impacts into medical education and professional responsibilities. In this study, Iranian medical students were assessed on their attitudes regarding climate change and whether it should be included in the curriculum.

**Methods:**

A national online survey was conducted at Smart University of Medical Sciences from November 2021 to March 2022. The participant group comprised 553 medical students from Iran who were enrolled in the summer semester program at Smart University of Medical Sciences (SMUMS), representing 38 medical universities across the country. The research instrument included 22 items on a 5-point Likert scale, focusing on climate change in medical education, adapted from two earlier questionnaires. Content validity was established through expert review using Content Validity Ratio (CVR) and Content Validity Index (CVI) methods, leading to a refined 22-item questionnaire. Face validity was confirmed via pilot testing with students. Reliability analysis with 100 participants showed satisfactory internal consistency (Cronbach’s alpha). It addressed two categories, which include six key components: curriculum enrichment, teaching activities, attention to learning, concern for others, social responsibility, and individual accountability. Data analysis was performed using SPSS version 24.

**Results:**

Out of 650 medical students who received the questionnaire, 553 completed and returned it, resulting in a response rate of 86%. The sample comprised 177 males (32%) and 376 females (68%). In the category of Integrated Education, the mean scores were as follows: Curriculum Enrichment (3.2), Teaching Activities (2.82), and Attention to Learning (3.01). For the Self-Responsibility category, the results included Concern for Others (3.63), Social Responsibility (3.52), and Individual Responsibility (3.78), with Individual Responsibility receiving the highest score. The independent t-test showed a statistical difference between gender and opinion toward climate change (*p* = 0.01). The analysis of variance showed that the differences between mean scores of academic years from various years of medical school were statistically significant (*p* = 0.03).

**Conclusions:**

According to the study, integrating climate change into medical curricula is essential to equip future physicians with the knowledge and responsibility to address climate-related health challenges.

## Introduction

 The world’s climate system is currently experiencing extraordinary changes. The number of extreme weather occurrences, such as rising temperatures, prolonged heatwaves, storms, floods, droughts, and wildfires, has steadily increased since the 1990s in every country in the world [1, 2]. The United Nations Framework Convention on Climate Change (UNFCCC) defines climate change as (CC) is ‘*the change that can be attributed directly or indirectly to human activity that alters the composition of the global atmosphere and which is in addition to natural climate variability observed over comparable time periods.’* [3]. The World Health Organization (WHO) recognized climate change as an existential threat to world health in the twenty-first century before the COVID-19 pandemic [4]. Climate change causes temperature-related sickness, harm, and mortality as well as flood and hurricane damage [5]. Numerous studies have highlighted the influence of global climate change on human well-being [6, 7]. A growing body of literature indicates that adapting strategies to mitigate climate change benefits public health [8–10]. Medical students are clearly distressed by the significant and complex challenges posed by climate change, including its impacts on public health, the healthcare system, and their future roles as healthcare professionals [11]. Medical students have a tremendous opportunity to demonstrate themselves as trustworthy advocates for global efforts to decrease emissions. This will protect populations from climate change hazards [12–15]. Threats to community health and well-being, including climate change, must be addressed in the medical education curriculum [14, 16, 17]. Healthcare providers are increasingly aware that medical schools need to prepare students to enter the medical profession with knowledge of how climate change influences their patients’ well-being and health [18–20]. Some studies focus on the lack of expertise, mindset, and awareness among medical students globally regarding climate change’s adverse impacts on people’s well-being [21, 22].

Health professionals play a vital role in shaping environmental health policies, building community resilience, and formulating adaptive strategies. Their understanding of climate-related issues is essential for meaningful engagement and successful outcomes in these areas. To address existing gaps in knowledge and practice, it is important to adopt strategic measures, such as identifying the specific climate change competencies required in healthcare and incorporating them into the education and training of health professionals. This approach can enhance their preparedness and effectiveness in responding to the health challenges posed by climate change [23].

It is expected that educating medical students about the negative effects of climate change and providing innovative approaches for dealing with them could bring proper opportunities for support of global initiatives to decrease emissions and support those who have been impacted by climate change trends globally [24, 25]. Other studies have revealed that although medical students know the adverse impacts of climate change on health, they are unprepared for mitigation and adaptation initiatives [25, 26]. Climate change requires medical schools to incorporate climate change effects into their curriculums [12, 16, 24]. The American Medical Association (AMA) has published a policy resolution for the instruction of climate change subjects throughout the medical education continuum with the objective of providing physicians with a foundational understanding of the subject matter [27]. Furthermore, by 2035, “climate-change medicine” will become a recognized specialty in internal medicine, according to the Accreditation Council for Graduate Medical Education (ACGME) [11]. The International Federation of Medical Student Associations (IFMSA) emphasizes health, the environment, and climate change worldwide [28].

Iran is located in West Asia and is the seventeenth largest country in the world, with a population of about 80 million. Iran is largely arid and semi-arid. Iran’s climate is diverse, with a wide range of terrestrial and marine environments [29]. Over the past few years, it has faced problems such as reduced rainfall, droughts, water scarcity, and pollution from urban and industrial activities. It has also faced desertification, soil erosion, and biodiversity loss [30]. Iran has an average rainfall of 240 millimeters per year [31]. Environment and Climate Change Canada(ECCC) has caused economic and social consequences, including urban migration, reduced agricultural production, and environmental degradation. As the temperature rises throughout Iran, especially in its central and southern regions, it is anticipated to increase by 4.5 degrees Celsius by 2100 [32]. Iran is the eighth largest carbon dioxide emitter after China, the United States, the European Union, India, the Russian Federation, Japan and Canada [33]. Education on climate change provides medical professionals with the essential knowledge and competencies to comprehend the intricate relationships between environmental factors and human health. Such education enhances their capacity to recognize health conditions sensitive to climate variations, advocate for sustainable practices, and engage in multidisciplinary collaborations aimed at mitigating climate-related health risks. Incorporating climate change education into medical curricula empowers future healthcare providers to bolster the resilience of health systems and improve community health literacy. Consequently, this integration supports public health preparedness, optimizes patient outcomes, and encourages active community participation in sustainability efforts [34, 35]. the medical curriculum in Iran primarily focuses on clinical subjects and basic sciences, traditionally giving less attention to environmental issues and climate change. However, it is essential to recognize that climate change significantly impacts public health and community well-being. Given the effects of climate change on diseases and public health, topics related to the environment and climate change must be integrated into medical education programs. As a result, Iran has engaged in extensive climate change research. However, to our knowledge, no studies have systematically examined Iranian medical students’ perceptions of climate change’s health implications. In addition, no studies have examined how the topic is included in the medical curriculum. Therefore, this article assesses Iranian medical students’ attitudes toward climate change and the inclusion of this topic in the medical curriculum. Adding Iranian medical students’ attitudes to the expanding body of literature on climate change knowledge and attitudes among health professionals is crucial.

## Methods

### Study design

In this nationwide descriptive cross-sectional study, the samples consisted of Iranian medical students who joined the summer semester program at Smart University of Medical Sciences (SMUMS) in Iran in 2022.

## Participants

The convenience sampling method was employed to select the sample. The summer semester programs included all basic and clinical science courses, where students (Medicine) had enrolled from 38 Iranian medical universities. The total number of registered students was 1,070; however, according to Cochran’s formula, a sample size of approximately 285 was deemed sufficient.

$$\:n=\frac{\frac{{Z}^{2}pq}{{d}^{2}}}{1+\frac{1}{N}(\frac{{Z}^{2}pq}{{d}^{2}}-1)}$$

Given the potential for sample attrition and low response rates with electronic questionnaires, we distributed the e-questionnaire link via email to 650 students.

### Instruments

Data were collected using a researcher-made questionnaire based on a modified of two questionnaires:

#### Q1. Climattitude questionnaire (12 Items)

The first is the ClimAttitude questionnaire. Bugaj et al. developed this scale in 2020 [24]. They developed this scale based on experts’ opinions of physicians and psychologists, and its validity was determined by expert validation. The questionnaire consists of 12 items and three dimensions, including knowledge of expected consequences, feelings of individual responsibility, and a sense of professional responsibility.

#### Q2. Climate change in medical curriculum (56 Items)

The other questionnaire was developed by Horton et al. In 2018 [36]. It aimed at assessing Australian medical students’ attitudes towards the inclusion of the topic of health impacts of climate change in the medical curriculum.

This questionnaire was developed based on a literature review and focus groups. It contains 56 items on the following areas: “Attitudes towards climate change, Health impacts of climate change for Australia and countries around the world, Roles of future doctors about health impacts of climate change, Roles of healthcare in addressing climate change through reductions in greenhouse gas emissions, and Attitudes about whether and How climate change should be incorporated into the medical school curriculum.”

#### Modified integrated questionnaire

The questionnaire was developed by adapting the two mentioned questionnaires. Following the guidelines set by the World Health Organization (WHO), both questionnaires were translated and retranslated and culturally adapted [37].

In adapting the Horton et al. (2018) tool, all 56 original items were examined with respect to the study objectives—namely, assessing medical students’ attitudes toward climate change education—and the cultural context of the target population. Items that were directly relevant, non-redundant, and contextually appropriate were retained, resulting in 15 items from the Horton tool. These items, together with 7 items adapted from the second instrument, constituted the final 22-item questionnaire, which was designed to ensure comprehensive coverage of relevant dimensions while minimizing respondent burden.

Following this customization, we re-evaluated the validity and reliability of the revised questionnaire. The final questionnaire was approved by experts, consisting of 22 items on a 5-point Likert scale (from Strongly Agree = 5, Agree = 4, Somewhat Agree = 3, Disagree = 2, to Strongly Disagree = 1). Considering the score range of 1 to 5, a cutoff point of 3 was established.

#### Content Validity

To assess content validity, the questionnaires were distributed to a focus group of 10 experts, comprising five Ph.D. holders in Medical Education, three clinical faculty members, and two Ph.D. holders in Basic Medical Sciences. Their feedback was incorporated into the revised versions of both questionnaires. The content validity was assessed using the Content Validity Ratio (CVR) and Content Validity Index (CVI) methods. In the Reliability Coefficient method, experts were asked to select essential options. Given that 10 specialists reviewed the validity, an agreement of at least 62% was expected. In the CVI method, specialists rated each item based on relevance, simplicity, and clarity (Scaled from 1 to 4). For calculating the CVI in each domain, only options rated 3 and 4 were counted, and the CVI was calculated according to the formula below. In this method, an agreement of approximately 79% for each item was anticipated. Overall, out of 56 items in the Climate Change in Medical Curriculum questionnaire, 41 items received low scores, and most options did not achieve sufficient ratings for relevance and simplicity. Additionally, 41 items were deemed somewhat useful by experts but were not essential; therefore, they were removed from the questionnaire, resulting in the selection of 15 items. The second questionnaire, the ClimAttitude Questionnaire, was evaluated, and out of 12 items, 11 were deemed suitable and received adequate scores, while one item was identified as unnecessary and irrelevant.

#### Face validity

Subsequently, in the second phase, a modified questionnaire with 26 questions was assessed with the participation of five medical and basic medical science students, focusing on its face validity. Ultimately, the final questionnaire with 22 questions was determined to be appropriate for measuring the research objectives.

It is noteworthy that in addition to assessing necessity, simplicity, structure, grammar of sentences, etc., the classification of items was also reviewed. Based on semantic similarities and content analysis of the items, the final questionnaire with 22 items was categorized into two categories: Self-Responsibility (related to individuals) and Integrated Education (requirements for change in education). Each of these categories includes components that are shown in Table 1. Overall, the CVI validity for each question in the final questionnaire was confirmed with values ranging from 0.80 to 1, and the CVR values ranged from 0.90 to 1.00.

#### Reliability

After distributing and collecting 100 samples, the reliability of the questionnaire was assessed using the method of internal consistency through Cronbach’s alpha, and the reliability of each domain was also evaluated separately (Table 1).

The overall Cronbach’s alpha coefficient for the final questionnaire was 0.965, indicating excellent internal consistency. The Cronbach’s alpha values for each domain are presented in Table 1 and ranged from 0.774 to 0.952, confirming acceptable to excellent reliability for all domains.

**Table 1:** The modified questionnaire of environmental challenges and climate change in medical education

Category	Concepts	Items	Reliability
Integrated Education	Curriculum enrichment	Q01-Q04	0.952
	Teaching activities	Q05-Q07	0.890
	Attention to learning	Q8-Q11	0.850
Self-Responsibility	Concern for others	Q12-Q14	0.774
	Social responsibility	Q15-Q18	0.875
	Individual responsibility	Q19-22	0.920
Total	----	22 Items	**0.965**

### Data analysis

A web-based survey was carried out. The survey was anonymous and voluntary, and informed consent was obtained from the respondents. Google Forms software was used to create the online survey. In the cover letter, the study’s purpose was explained to the participants. They were asked to provide demographic information, including gender, age, and academic year in which they were studying. The data were analyzed using frequency counts, percentages, a t-test, and an Analysis of Variance. The data were analyzed using the statistical software package IBM SPSS Statistics V 24, and a p-value of less than 0.05 was considered significant.

### Ethics

Smart University of Medical Sciences approved this research through the research ethics committee with the registration number IR.VUMS.REC.1402.004. The questionnaires were distributed anonymously to the students, and the research objectives were communicated to them in the introduction of the questionnaire. Participants completed the questionnaires with informed written consent.

## Results

### Demographic characteristics

Of the 650 medical students who received the questionnaire, 553 completed it (86%) and returned it from 38 medical schools. The sample included 177 males [32%] and 376 females [68%]. Table 2 shows the demographic data of the respondents.

**Table 2:** Demographic characteristics of the respondents

Groups	*N* (%)
Gender	
Male	177 (32%)
Female	376 (68%)
Academic year	
1st year	89 (16%)
2nd year	78 (14%)
3rd year	101(18%)
4th year	99 (18%)
5th year	60 (11%)
6th year	76 (14%)
7th year	50 (09%)

### Students’ perspectives on climate change in medical education

Table 3 presents the mean and standard deviation of participants’ perspectives on each item in the questionnaire, categorized into six concepts and two categories (see Table 3).

**Table 3:** Opinions of students regarding Items and concepts of the status of Climate Change in Medical Education

Concepts	Items	Mean ± St.D	
Category 1: Integrated Education		3.01	0.19
Curriculum enrichment	1) Medical degree programs should include educational activities focused on the health effects of climate change	3.43	0.63
	2) The health effects of climate change should be included in our existing medical curriculum.	3.65	0.22
	3) Some public health courses will be more engaging as a result of the impact of climate change.	3.24	0.57
	4) It would be ideal to incorporate courses on climate change early in the curriculum.	2.46	0.21
Teaching activities	5) It is essential for medical educators to emphasize the importance of climate change topics across various courses	3.01	0.58
	6) The importance of climate change should be emphasized in face-to-face teaching and educational activities.	2.69	0.23
	7) Topics related to climate change can be effectively taught through online learning.	2.75	0.35
Attention to learning	8) I believe that there should be an emphasis on learning and investigating diseases related to climate change	2.85	0.27
	9) Familiarity with the consequences of climate change compels me to critical thinking and analysis of related diseases.	3.17	0.29
	10) Medical students need to learn how to help people adapt to climate change.	3.45	0.80
	11) I am not worried that incorporating climate change into medical education will result in overlapping content.	2.56	0.17
Category 2: Self-Responsibility		**3.64**	**0.13**
Concern for others	12) I expect that climate change will have a detrimental impact on people’s health	3.63	0.46
	13) I expect that climate change will adversely affect my patients’ health shortly.	3.76	0.73
	14) I predict that climate change will increase infectious disease prevalence.	3.50	0.78
Social responsibility	15) I have a sense of social responsibility for climate change.	3.50	0.76
	16) I serve as a social role model for others regarding climate change matters.	3.52	0.58
	17) I am responsible for educating the public about the impacts of climate change	3.57	0.97
	18) I inform my patients about climate change.	3.50	0.90
Individual responsibility	19) I serve as a role model for my patients regarding climate change.	4.15	1.09
	20) I strive to be proactive in preserving the environment and non-renewable resources.	2.85	0.37
	21) I try to reduce air pollution by using public transportation.	4.17	0.89
	22) Due to environmental challenges and climate change, I reduce my plastic waste.	3.93	0.77

**Fig. 1:**
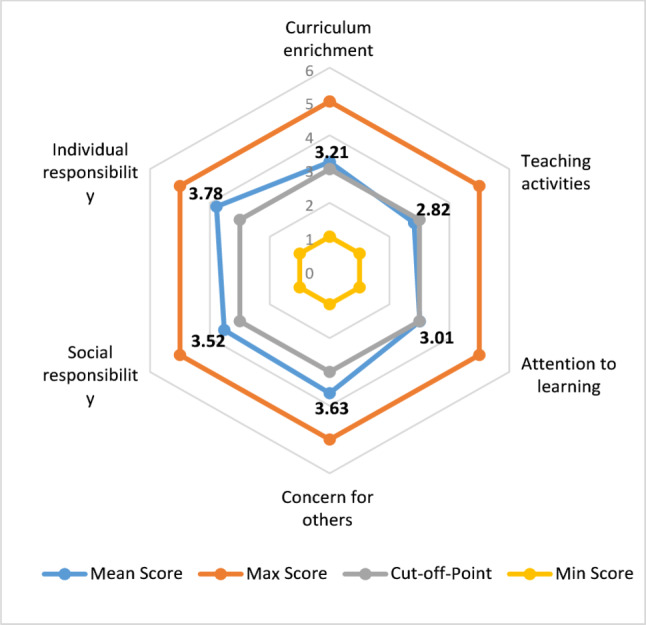
The average of the components of the questionnaire on climate change in medical education

According to Table 3, the necessity of paying attention to climate change in medical education has been emphasized from the perspective of students, and both categories of factors related to Integrated Education and Self-Responsibility have scored above 3, with the score for Self-Responsibility being reported higher. Additionally, the score for each component of the questionnaire is shown in Fig. 1. Among the components, the highest average is related to individual responsibility (m = 3.78) (Fig. 1).

### Students’ perspectives on contextual variables

Gender: As shown in Table 4, the independent t-test showed a statistical difference between gender [t = 5.57, *p* = 0.01] and opinion toward climate change. The mean score was higher for female participants (*N* = 376; M = 3.85, SD = 0.95) than for male participants in their opinions toward climate change. The independent t-test confirmed that this difference was statistically significant, indicating that female participants scored significantly higher than male participants.

#### Academic years

The analysis of variance revealed that the differences in mean scores across academic years were statistically significant [F = 3.09, *p* = 0.03]. The results suggest that students in more advanced years place greater emphasis on the necessity of integrating climate-related topics into medical science curricula. This trend reflects a growing awareness among senior students regarding the relevance of environmental issues and climate change to their future medical practice (see Table 4).

As they progress in their studies, these students recognize the importance of being equipped with knowledge and skills to address the health impacts associated with climate change, thereby advocating for its inclusion in medical education.

**Table 4:** Group comparison scores of the questionnaire

Groups	*N*	Mean	SD	Statistical Significance
Gender				
• Male	177	2.91	1.02	*p* = 0.01 t = 5.47
• Female	376	3.85	0.95	
Academic year				
• 1st year	89	2.89	0.99	*p* = 0.03 F = 3.09
• 2nd year	78	2.97	1.12	
• 3rd year	101	3.01	0.83	
• 4th year	99	3.48	0.67	
• 5th year	60	3.42	0.83	
• 6th year	76	3.81	0.88	
• 7th year	50	3.98	0.95	

## Discussion

This survey, the first in Iran, assessed medical students’ attitudes toward climate change. It provides insight into the perspectives of Iranian future health workers regarding climate change. As the negative effects of climate change become increasingly evident, medical students increasingly acknowledge the importance of addressing this issue effectively. Medical students demand further climate change education to prepare for future practice in a rapidly changing environment [13].

This study indicates that despite their interest in climate change topics, medical students expressed a need for improved education on this subject. Also, medical students reported that curricula should focus on climate change health impacts. They need to learn how to help people adapt to climate change. This result is consistent with other studies that indicate that medical students are aware of climate change’s health effects but feel unprepared for health sector mitigation and adaptation [14, 16, 25, 28, 38]. From the perspective of medical students, they recognize the importance of having knowledge of local climate health threats. As evidence increasingly shows that climate system changes can negatively impact human health, medical school curricula need to include content addressing the health risks and harms linked to climate change. A survey conducted in 2013 revealed that 34–40% of medical students graduating between 2009 and 2013 felt their education in environmental health was insufficient [17]. The urgency of these findings grows as we gain a deeper understanding of the potential dangers posed by climate change to both the environment and human health. Findings suggest a perceived need for improved efforts in medical schools to equip students with the knowledge and awareness of how climate change adversely affects human health. There is no doubt that climate change is a topic to include in medical curricula. Given the findings, it seems that adding a course to the curriculum may lead to curriculum overlap, which Abrahamson mentions as Curriculum Hypertrophy [39]. Therefore, it is suggested that concepts related to climate change and its role in health be integrated into all subjects based on a nested integrated curriculum approach. The nested integrated learning model refers to the integration of curriculum elements within a single discipline, focusing specifically on addressing various learning skills simultaneously [40]. It may be more effective and efficient to incorporate eco-medical literacy and environmental literacy as part of the curriculum and teaching than to argue that medical education needs to modify an already overloaded curriculum [19]. This approach enables a thorough comprehension of the effects of climate change on health without requiring the introduction of completely new courses. Moreover, integrating modules promotes interdisciplinary learning and encourages students to consider health issues within a wider ecological context. This strategy holds particular significance in Iran, where environmental challenges such as air pollution and water scarcity represent critical public health issues.

Despite efforts to integrate climate change education in Iran, several challenges hinder its effective implementation. The existing curriculum is highly centralized, restricting the adaptability needed for innovative and flexible teaching approaches. Moreover, there are significant resource constraints, including insufficient training opportunities for educators and a lack of educational materials focused on climate change. Cultural resistance to moving away from traditional teaching methods further complicates these efforts [41]. To address these obstacles, policy reforms should focus on creating a more flexible and interdisciplinary curriculum. Additionally, investing in professional development programs is crucial to equip educators with the skills required to effectively deliver integrated and updated content. Environmental literacy is an extension of occupational and environmental medicine [42]. Medical students must also understand the ‘one health approach,’ in which animal, environmental, and human health are all interconnected [43]. Wellberry et al. give examples of how to include climate change in medical school curricula at all levels of medical education. Several institutions are also considering long-term, integrated curricula for climate change [22, 44].

analysis revealed that students placed greater emphasis on the inclusion of the subject matter across all courses, while the instructional approach —defined in this study as the methods and formats used to deliver educational content, including face-to-face lectures, online learning, and course sequencing—was considered less critical. This finding is further supported by the lower scores assigned to items 4, 6, and 7, which focused on teaching activities. These results suggest that, from the students’ perspective, content integration holds greater significance than the specific methods of instruction. One possible explanation for the marginal score observed in the “Integrated Education” category relates to students’ perceptions regarding the relative importance of content versus teaching methods.

The findings of this study showed that regarding the opinion of the medical students about the sense of Self-Responsibility to climate change, the medical students agreed that they function as social role models about climate change for patients and they are responsible for educating the public about the impacts of climate change. Possible explanations include medical students playing a crucial role in reducing climate change’s health impacts. Climate change adaptation, education, and mitigation programs will be part of their professional responsibilities [12, 25, 45]. Those findings are consistent with Bugaj’s findings that medical students agreed with their roles as social role models and role models for patients in climate change [25]. However, fewer students recognized that they had a social responsibility, educational role, and informational role in climate change. The majority of medical students recognize the significant role they play in developing tomorrow’s doctors; however, they do not translate that responsibility into direct actions, such as educating patients about climate change.

In this study, the Individual Responsibility component received the highest score and the majority of medical students believed that as individual responsibility they try to reduce air pollution and limit plastic garbage use. This result is imperative because medical students are front-line workers in the climate crisis [11]. Teaching sustainable medicine and promoting transformation to sustainable healthcare requires students and clinicians to learn from each other [46]. Furthermore, as leaders and medical experts, medical students play a significant role in advocating for climate change [11].

In this study, a majority of medical students believed that climate change would increase infectious disease prevalence in the near future. They predicted that it would harm their patient’s health soon, according to this study. This finding is in agreement with Yang et al. findings which showed that 83% of medical students believe climate change is generally harmful to humans. They all anticipate increased local, national, and international consequences of climate change shortly [47]. The attitudes of medical students in this study appear to be similar to those of Hampshire et al. who found that most U.S. medical students believed climate change would affect population health outcomes in the study. This will affect their health, as well as their patients in the future [16]. Physicians need to know how climate change impacts clinical practice. In the study of Nigatu et al. over three-quarters of health sciences students were aware of the health consequences of climate change [48]. Hathaway et al. found that it appears that health professionals perceive climate change as having negative health effects, but their knowledge is low. They perceive a need to learn more about it [49]. Research conducted by Boland et al. revealed that 64% of physicians believe climate change affects their patients’ health and 17% are comfortable counseling patients about climate change [50]. These results may be explained by the fact that 23% of deaths and 22% of disability-adjusted life-years (DALYs) worldwide are attributed to environmental risk, and nearly a quarter of the global disease burden could be reduced by reducing environmental risks [51]. It is also imperative to note that rising temperatures have implications for vector-borne and water-borne diseases, both of which influence infectious disease spread [52]. According to a literature review published in 2018, most health professionals understand that climate change is occurring and affects their patients’ health; however, they still think they lack sufficient knowledge [49].

The findings of this study indicate a meaningful difference between men and women in their attitudes toward climate change, with women demonstrating a more positive or sensitive outlook on this issue. This result is consistent with previous research suggesting that women generally express greater concern about environmental issues and are more likely than men to support environmentally friendly policies and behaviors [53–56]. Such differences may stem from various factors, including differences in socialization, risk perception, and values related to environmental protection. However, a study from India reported the opposite pattern, with male students demonstrating greater awareness than female students. Additionally, research from the Arab region found no significant relationship between gender and awareness of climate change’s health consequences [57]. Overall, most studies suggest that gender plays a significant role in shaping students’ awareness of the health impacts of climate change. Recognizing these gender-based differences can help in designing more effective interventions and communication strategies to raise awareness and encourage engagement with climate change issues.

Additionally, the analysis revealed statistically significant differences in mean scores across various academic years in medical school. Students in the later years of medical school tend to place more importance on incorporating climate-related topics into their medical education. This finding aligns with previous research showing that as students advance in their education, their knowledge and appreciation of climate-related health issues tend to increase [53].

This pattern likely reflects a growing awareness among more advanced students of how environmental issues and climate change are relevant to their future roles as healthcare professionals. As students progress through their training, they may better appreciate the impact of climate change on health outcomes and the need for medical curricula to address these emerging challenges. Undergraduate medical education curriculums addressing climate change foster critical thinking, participation in sustainable global initiatives, multidisciplinary perspectives, and public health literacy - all crucial capabilities for physicians in a rapidly changing environment [44]. In order to achieve a climate-educated physician workforce capable of addressing the challenges of the healthcare system and the health industry, education and training must be aligned with workforce considerations and climate action [58].

### Strengths and limitations

The present study was conducted with a large sample of medical students from universities across the country, and therefore, the results reflect a diversity of perspectives. Additionally, the instrument developed from this research, which has been evaluated for content validity by experts and has high reliability, can also be used in other studies. However, this instrument has been used for the first time, and thus it is necessary to re-test and validate it in future research.

The environment and its challenges are a global issue, while also being a regional and climate-dependent topic. Given the climate-related health issues and challenges in Iran, a comprehensive examination of this subject is necessary. In this study, we have focused on the perspectives of one stakeholder (students), and it is recommended that future research explore these challenges from the viewpoints of other stakeholders as well. also, one of the limitations of this study is the use of convenience sampling, which may introduce selection bias and limit the generalizability of the findings to the broader target population. Therefore, the results should be interpreted with caution. Future research is recommended to utilize more rigorous sampling techniques to enhance representativeness and the validity of findings.

## Conclusion

This survey provides valuable insight into the attitudes of medical students toward climate change and its integration into medical education. The findings reveal that most students recognize the significant health impacts of climate change and strongly support incorporating related topics into the medical curriculum. Notably, female students and those in higher academic years demonstrated significantly more positive attitudes toward the inclusion of climate change and its health consequences in medical education. While students expressed a keen interest in climate change, they also reported a perceived gap in their formal education and a lack of preparedness to address climate-related health challenges in their future practice. These results highlight the need for Iranian medical schools to enhance their curricula by integrating climate change and environmental health concepts throughout all years and subjects, rather than as a standalone course. Such an approach will better equip future physicians with the knowledge, skills, and sense of responsibility needed to address the growing health challenges posed by climate change. By integrating curriculum changes and advocacy training, medical students will be better equipped and inspired to succeed in their future careers.

## Data Availability

The authors confirm that the data supporting this study’s findings are available in the article and its supplementary materials.
